# The Story of the Insane from Year to Year

**Published:** 1901-12-21

**Authors:** 


### THE STORY OF THE INSANE FROM YEAR TO YEAR.

(Thirteenth Series.)

### BELFAST DISTRICT ASYLUM.

The limits of accommodation are stated to be 400 beds, but the asylum contained 907 patients on December 31st, 1899. If this means that the asylum contains more than twice as many as it was intended for, there is something radically wrong, and someone greatly to blame. There were 288 first admissions, 46 readmissions, 100 recoveries, 40 discharged relieved, and 79 deaths. The number of persons under care during the year was 1,314, and the daily average number resident was 922. Calculated on the admissions, the recoveries stand at 29 9 per cent.; and calculated on the average number resident, the deaths stand at 8·6. Of the deaths, 35 were caused by cerebral and spinal diseases and 12 by consumption. Of the admissions 10 owed their insanity to domestic trouble, 8 to adverse circumstances; 9 to religion, 2 to love, 20 to intemperance in drink, and 38 to hereditary influence. It is proposed to build a new asylum large enough to accommodate about 820 patients; and it would seem from the report that the original idea was to arrange the buildings somewhat after the style of the modern district asylums of Scotland. Broadly speaking, that means that it was to have had one section for the acute, the sick, and the infirm, and another section for the chronic patients. This system, whatever theoretical objections may be urged against it, has certainly borne the test of considerable experience, whereas the plan now adopted can hardly be said to have any life at all in England as yet. Furthermore, we look upon the striking out of the small dining-rooms and their substitution by one large one as a distinctly retrograde step, and one which cannot fail to be the cause of much unpleasantness to the poor patients for whose welfare the asylum is built. It is much to be regretted that the Belfast committee did not consult one of the Scottish experts in asylum construction (say Sir John Sibbald) before finally deciding the more important points of their new asylum. Judging from the figures in this, the 70th annual report of the institution, we are further of opinion that the number of beds will soon prove to be insufficient for such a city as Belfast. The annual cost per head was £24 3s. 4d.

